# Metagenome sequencing and 103 microbial genomes from ballast water and sediments

**DOI:** 10.1038/s41597-023-02447-x

**Published:** 2023-08-10

**Authors:** Zhaozhao Xue, Yangchun Han, Wen Tian, Wei Zhang

**Affiliations:** 1 Marine College, Shandong University, Weihai, 264209 China; 2 Integrated Technical Service Center of Jiangyin Customs, Jiangyin, 214441 China; 3 Animal, Plant and Food Inspection Center of Nanjing Customs District, Nanjing, 210001 China

**Keywords:** Water microbiology, Marine biology

## Abstract

The great threat of microbes carried by ballast water calls for figuring out the species composition of the ballast-tank microbial community, where the dark, cold, and anoxic tank environment might select special taxa. In this study, we reconstructed 103 metagenome-assembled genomes (MAGs), including 102 bacteria and one archaea, from four vessels on international voyages. Of these MAGs, 60 were ‘near complete’ (completeness >90%), 34 were >80% complete, and nine were >75% complete. Phylogenomic analysis revealed that over 70% (n = 74) of these MAGs represented new taxa at different taxonomical levels, including one order, three families, 12 genera, and 58 species. The species composition of these MAGs was most consistent with the previous reports, with the most abundant phyla being Proteobacteria (n = 69), Bacteroidota (n = 17), and Actinobacteriota (n = 7). These draft genomes provided novel data on species diversity and function in the ballast-tank microbial community, which will facilitate ballast water and sediments management.

## Background & Summary

Ballast water is routinely used to maintain the ship’s balance and safety throughout the voyage. With the rapid globalization of trade, it is estimated that each year over 10 billion tons of ballast water are transferred worldwide^1^. Accompanying, many harmful non-indigenous species (NIS) carried by ballast water have caused serious threats to ecological and human health^2,3^, among which a well-known example was the international dissemination of *Vibrio cholerae*^4,5^. Therefore, a comprehensive insight into the diversity and distribution patterns of microbial communities in ballast water is crucial to ballast water management (BWM).

The development of high-throughput sequencing skips the necessity of microbe culture and allows a large number of unknown taxa to be discovered^6,7^. In recent years, the microbial diversity of ballast water and its sediments has been largely investigated by 16S rDNA amplicon sequencing^2,3,8,9^. However, amplicon analysis using one or a few gene regions often fails to distinguish closely related species when assessing community diversity. Alternatively, metagenomics provides abundant gene information about microbes through high-throughput sequencing, and the assembly of these genes could identify a large number of uncultured microbes^10^. With the advances in metagenomic sequencing, over 14,000 microbes have already been identified from complex samples of ballast water and sediments without cultivation, revealing the hidden microbial diversity in ballast water and sediments^11^. In this study, we further demonstrated this hidden microbial diversity by retrieving and assembling their metagenomic sequences into near complete microbial genomes, because metagenome-assembled genomes (MAGs) can provide more accurate information about microbial species and their communities^12,13^.

**Fig. 1:**
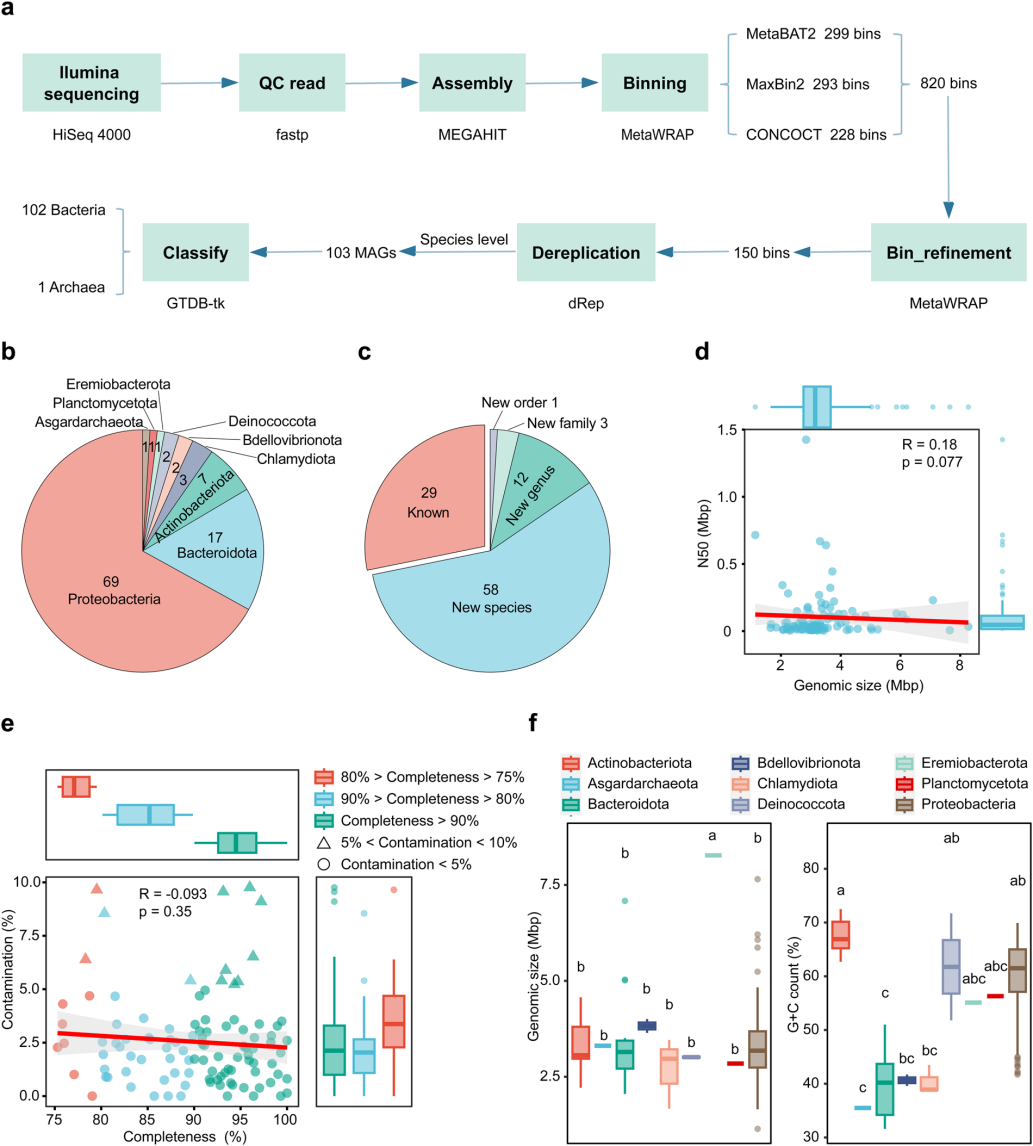
Overview of the MAGs. (**a**) The workflow of MAG reconstruction. A bolded font represents the key processes, and directly below are the tools implemented. (**b**) The distribution of all MAGs at the phylum level. (**c**) Potential taxonomic novelty of MAGs at different taxonomical levels. (**d**) The relationship between genomic size and N50 length among MAGs. (**e**) The relationship between the completeness and contamination of MAGs. (**f**) Boxplots compare the distribution of genomic size and GC content among MAGs at the phylum level. Boxplots of MAG that do not share any lowercase letters (**a**–**c**) indicate that they are significantly different (P < 0.05).

We successfully reconstructed 103 MAGs by collecting samples of ballast water and sediment from four international vessels (Table S1; Fig. 1a–c). All of these MAGs have a completeness of >75% with a contamination <10% (Table S2). In other words, all of the 103 MAGs meet the medium quality of the MIMAG standards^14^. Of these MAGs, 60 (58%) were ‘near complete’ (completeness >90%), 34 (33%) were >80% completeness, and nine (9%) were >75% completeness (Table S2). In addition, 91 (88%) MAGs had <5% contamination, and 7(7%) MAGs had no contamination at all (Tables S2, S3). A total of 90 (87.38%) MAGs had a N50 length greater than 10,000 bp, with the longest value of 1.43 Mbp (Table S3), indicating excellent assembly quality. The genome size that was calculated from MAG completeness using CheckM v1.2.2^15^, ranged from 1.14 to 8.27 Mbp, with an average value of 3.38 Mbp (Table S3). At the phylum level, Actinobacteriota had the highest GC content (average 67.54%), in contrast, Asgardarchaeota of Archaea had the lowest GC content (35.50%, Tables S3, S4). There was no significant correlation between genome size and N50 length (Fig. 1d). Of all the MAGs, there was no correlation between their completeness and contamination, despite the fact that MAGs with much lower completeness (completeness <80%) usually had higher contamination (Table S3; Fig. 1e).

**Fig. 2:**
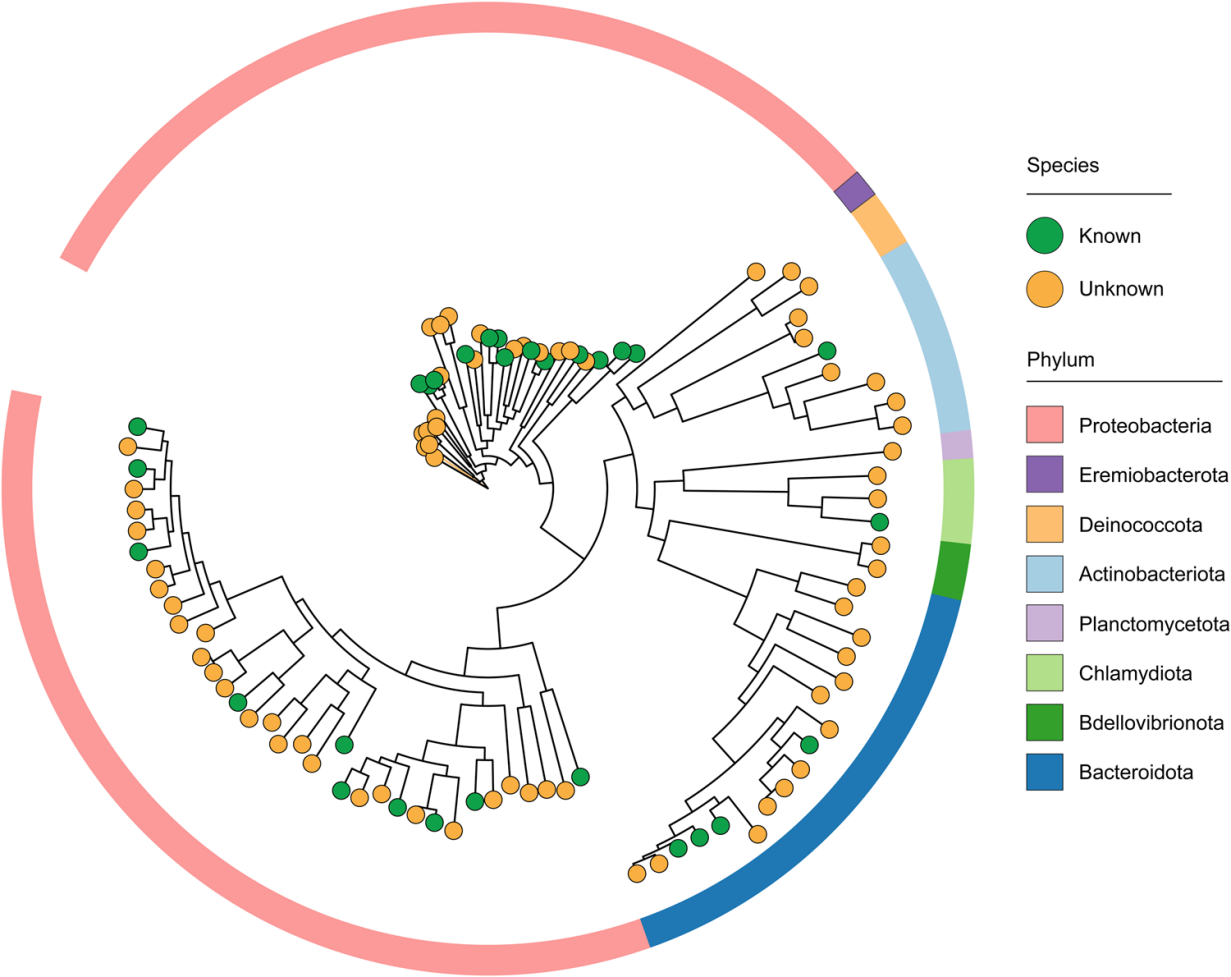
A phylogenetic tree of all species-level bacterial MAGs (n = 102) constructed from 120 conserved bacterial marker genes. The circle colors at the ends of the phylogenetic branches represent known species (green) and unknown species (orange) in GTDB. Different phyla of these MAGs were colored in the outermost ring.

According to the Genome Taxonomy Database (GTDB)^16^, these draft genomes were classified into 102 bacteria and 1 archaea (Fig. 1b). A total of nine phyla were identified; the most abundant phyla were Proteobacteria (n = 69), Bacteroidota (n = 17), and Actinobacteriota (n = 7; Figs. 1b, 2). Notably, 74 (71.84%) MAGs cannot be assigned to any named entry in GTDB, indicating that most of these MAGs represent novel taxa (Table S4; Fig. 2). In sum, one order, three families, 12 genera, and 58 species (57 bacteria and 1 archaea) were novel taxa (Table S4; Fig. 1c).

**Fig. 3:**
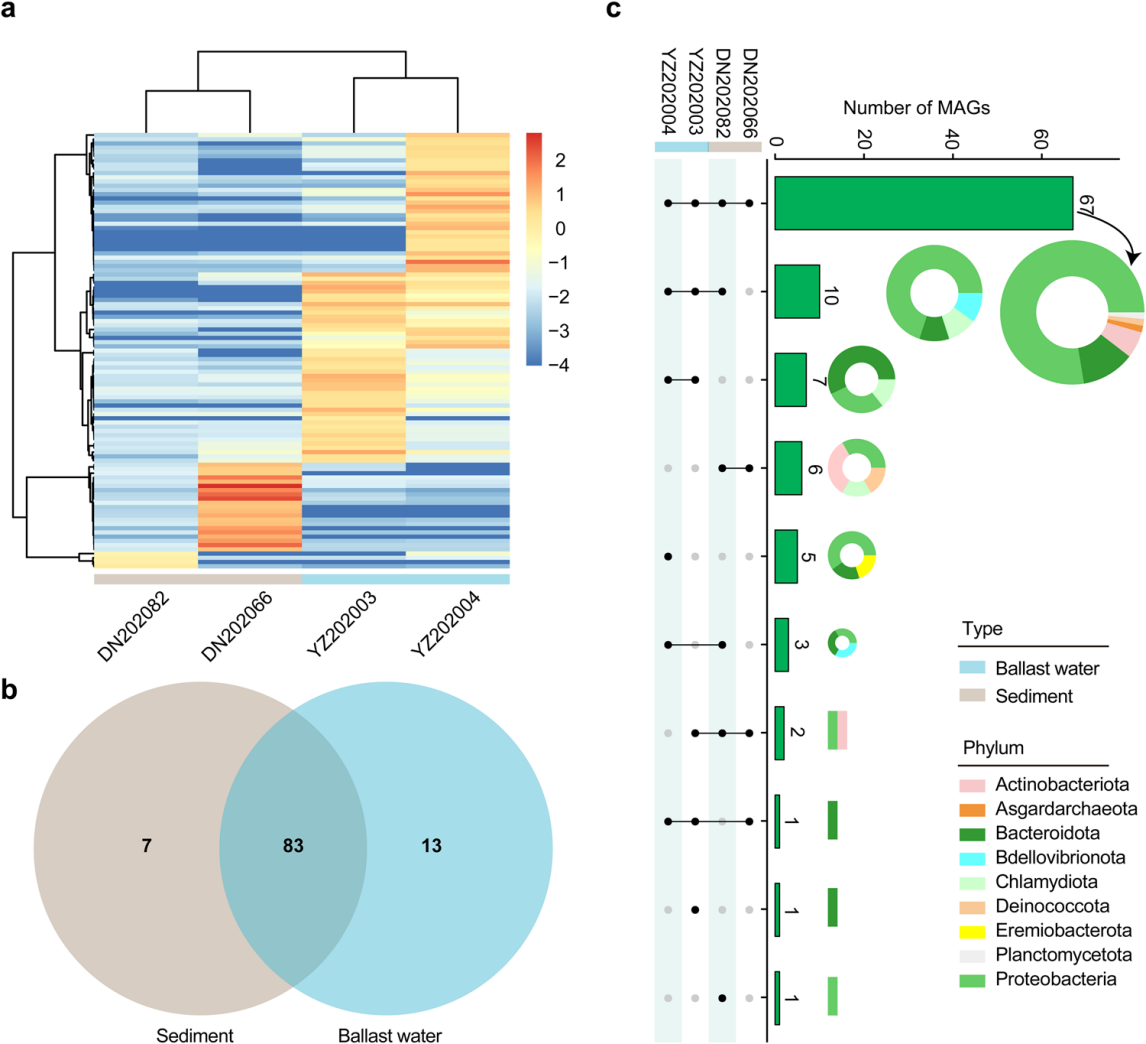
The distribution of the 103 MAGs among the four samples of ballast water and sediment. (**a**) A heatmap shows the MAGs and their relative abundances among samples. The relative abundances of MAGs were calculated by the MetaWRAP Quant_bins module, and were transformed into the positive/negative values by using the logarithmic transformation (log_10_). (**b**) A venn diagram shows the number of shared MAGs between ballast water and sediment. (**c**) The shared or unique MAGs across different samples. The histogram shows the number of shared MAGs among different sample combinations, and the colored rings/stacked bar plots show their different taxonomic compositions at the phylum level.

The abundance of these MAGs varied among different samples; in general, sediment samples had more MAGs than ballast water (Fig. 3a). There were 83 (80%) MAGs common in both ballast water and sediments (Fig. 3b). Moreover, 65.05% (67/103) of MAGs were shared by all samples, among which the Proteobacteria was the mainly shared phylum (52/67, Table S5; Fig. 3c).

To our best knowledge, this is the first study to recover microbial genomes separately from both ballast water and sediment samples. The repertoire of such microbial genomes from vessel ballast water and sediment can further facilitate the understanding of the species diversity, structure, and function of these microbial communities, which will greatly contribute to ballast water and sediments management.

## Methods

### Sampling and metagenomic sequencing

The techniques of collecting ballast water and sediment samples, as well as performing metagenomic sequencing, have been previously described^11^. Briefly, we collected ballast water samples from two vessels engaged in international voyages at the Jiangyin port in Jiangsu, China. Additionally, we obtained two sediment samples, each weighing approximately 500 g, from the ballast tanks during repair work at the shipyard. More detailed information about the sample collection can be found in Table S1. We extracted the total genomic DNA from the ballast water and sediment samples using the E.Z.N.A. Soil DNA Kit (Omega Bio-tek, USA) following the manufacturer’s instructions. The paired-end sequencing was performed on the Illumina HiSeq. 4000 platform (Illumina Inc., San Diego, CA, USA) at Majorbio Bio-Pharm Technology Co., Ltd. (Shanghai, China), resulting in the generation of 12 Gb of sequences per sample. The raw data can be accessed at the NCBI Sequence Read Archive (SRA) with the identifier SRP423788. The accession numbers for these data range from SRR23576959 to SRR23576962^17^.

### Quality control and assembly

The adapter sequences were removed, and the low-quality reads (length less than 15 bp, average quality score less than 15, or containing more than five N bases) were filtered by using fastp v0.21.0^18^ (parameters: default). Then all of the quality-controlled reads were co-assembled with MEGAHIT v1.2.9^19^ (parameters: default). The quality of the assembly was assessed using QUAST v5.0.2^20^.

### Genome binning, refinement, and dereplication

Based on tetranucleotide frequencies, coverage, and GC content, genome bins were recovered using the MetaWRAP v1.3.2^21^ pipeline (parameters: default), including MaxBin 2.0^22^, metaBAT 2.0^23^ and CONCOCT v1.0.0^24^ metagenomic binning software. The binning results (820 bins) were refined using the MetaWRAP-Bin_refinement module (parameters: -c 50 -x 10), and 150 bins were finally obtained. A lineage-specific work flow of CheckM was used to estimate the completeness and contamination of these genome bins. The bins were then quantified by using the MetaWRAP-Quant_bins module of MetaWRAP (parameters: default). The refinement bins were dereplicated using dRep v2.6.2^25^ (parameters: -sa 0.95 -nc 0.30 -comp 50 -con 10) at the 95% average nucleotide identity (ANI), resulting in 103 unreplicated species-level MAGs.

### Taxonomic classification and phylogenetic analysis of MAGs

The classification of MAGs was performed by the classify_wf workflow of GTDB-TK v2.0.0^26^ with GTDB release 207 (parameters: default). A phylogenetic tree of 102 species-level bacterial MAGs was constructed by 120 bacterial marker genes using the gtdbtk infer module in GTDB-TK (parameters: default). The tree was annotated and visualized by iTOL v5^27^.

## Data Records

The 103 species-level MAGs have been submitted to DDBJ/ENA/GenBank^28–130^ and figshare^131^.

## Technical Validation

To avoid contamination of samples, all sampling tools and containers have been sterilized before sampling. After the samples were obtained, they were immediately placed on ice and kept away from light, and then sent to the laboratory within two hours for further processing to ensure the quality of the DNA. The distribution size of the fragmented DNA and the amplified library was characterized using the Agilent 4200 TapeStation system. Size selection of the fragmented DNA and the amplified library was performed by SPRI cleanup and the BluePippin instrument. Quantification of the pooled library using quantitative PCR. The completeness and contamination of the draft genomes were validated using CheckM.

### Supplementary information

supplementary information

## Data Availability

Custom scripts were not used to generate or process this dataset. Software versions and non-default parameters used have been appropriately specified where required.
