# Supplementary material for: Metagenome sequencing and 103 microbial genomes from ballast water and sediments
Source: Sci Data. 2023 Aug 10;10:536. doi: 10.1038/s41597-023-02447-x (PMC10415351; doi:10.1038/s41597-023-02447-x)
Supplement: Supplementary file 1 — supplementary information [file 41597_2023_2447_MOESM1_ESM.pdf]

Supplementary Information

Metagenome sequencing and 103 microbial genomes from ballast water and sediments

Zhaozhao Xue[1], Yangchun Han[2], Wen Tian[3], Wei Zhang[1]✉

1. Marine College, Shandong University, Weihai, 264209, China
2. Integrated Technical Service Center of Jiangyin Customs, Jiangyin, 214441, China
3. Animal, Plant and Food Inspection Center of Nanjing Customs District, Nanjing, 210001, China

Corresponding author: Wei Zhang (wzhang@sdu.edu.cn)

Table of Contents

Table S1 The sampling information of ballast water and sediment

Table S2 An overview of the completeness and contamination of 103 MAGs

Table S3 The statistical information of 103 MAGs

Table S4 The classification of MAGs according to Genome Taxonomy Database

Table S5 The number and classification of MAGs across different sample compositions

Table S1 The sampling information of ballast water and sediment

Sample ID	Ship name	Sample type	Sampling date	Source of last BW uptake	Salinity	Turbidity
YZ202003	ASIAN PIONEER	Ballast water	2020-07-27	Open-ocean	30.9‰	0.5 (NTU)
YZ202004	THE DIPL OMAT	Ballast water	2020-07-29	AUSTRALIA	33.2‰	0.6 (NTU)
DN202066	ERNA OLDENDORFF	Sediment	2020-05-09			
DN202082	BW FALCON	Sediment	2020-06-03			

Table S2 An overview of the completeness and contamination of 103 MAGs

Completeness(Com)	Contamination(Con)		Total
	Con <5%	5% < Con <10%	
Com >90%	52	8	60
90%> Com >80%	32	2	34
80%> Com >75%	7	2	9
Total	91	12	103

Table S3 The statistical information of 103 MAGs

MAG	Completeness (%)	Contamination (%)	Genomic size (bp)	GC content (%)	Number of contigs	Maximum contig length (bp)	Contig N50 (bp)	Mean contig length (bp)
**MAG.1**	95.56	2.27	2898333	64.98	30	343536	179747	96611
**MAG.10**	97.22	9.1	8270751	55.19	398	119139	32778	20781
**MAG.101**	100	0.81	3277532	43.81	39	452789	269040	84039
**MAG.102**	89.44	1.08	3362417	59.07	177	128186	32477	18997
**MAG.103**	87.34	3.14	2905931	45.45	397	31508	8672	7320
**MAG.104**	98.32	2.82	4166386	72.56	88	362041	66963	47345
**MAG.105**	79.52	9.65	3880437	68.17	175	137154	35207	22174
**MAG.106**	95.91	1.68	4828010	67.07	69	258491	114433	69971
**MAG.11**	92.89	0.92	3311419	62.06	88	155004	57220	37630
**MAG.113**	93.58	0	1669687	43.50	53	119368	52693	31504
**MAG.114**	92.8	0.51	3038895	71.77	102	169888	42595	29793
**MAG.115**	80.34	8.55	3438669	64.02	953	58210	4690	3608
**MAG.116**	79.03	0	2051784	35.70	9	453431	342062	227976
**MAG.117**	94.72	5.36	4002760	39.66	140	248940	58265	28591
**MAG.119**	92.52	0.76	2759533	62.52	87	147847	55448	31719
**MAG.12**	80.37	1.25	3112133	66.15	57	237339	93958	54599
**MAG.120**	96.59	1.14	2843240	56.31	11	1425075	1425075	258476
**MAG.121**	96.39	0	3651156	57.19	30	651080	322477	121705
**MAG.122**	81.67	3.36	2527791	34.27	160	72258	22095	15799
**MAG.123**	93.46	3.27	3308062	35.53	12	995517	669118	275672
**MAG.125**	99.03	1.02	6071148	66.80	89	656023	122042	68215
**MAG.126**	87.41	1.76	5867786	66.26	69	339159	131510	85040
**MAG.129**	81.49	4.67	3375153	33.54	626	49086	8018	5392
**MAG.13**	94.32	5.23	3060676	66.94	406	49118	10584	7539
**MAG.130**	80.14	2.53	2307622	31.85	646	18975	4442	3572
**MAG.131**	88.04	2.52	2963450	70.47	448	43681	9223	6615

**MAG.132**	95.82	1.93	5010515	39.75	325	99492	25971	15417
**MAG.133**	75.83	3.37	2941581	65.15	86	210549	51945	34204
**MAG.134**	80.7	0.88	4295639	62.78	421	39255	14230	10203
**MAG.135**	91.1	2.37	3224272	62.92	239	60712	19477	13491
**MAG.136**	77.08	1.01	2082267	56.97	235	71592	12344	8861
**MAG.138**	96.4	1.55	3219460	60.31	135	110662	39392	23848
**MAG.14**	94.13	0.52	2696019	59.47	251	58665	14274	10741
**MAG.140**	96.35	2.22	3348430	61.07	25	530355	211473	133937
**MAG.143**	80.22	1.95	2519290	67.74	248	62370	13015	10158
**MAG.144**	94.31	3.57	2678600	50.83	44	223360	84480	60877
**MAG.145**	96.49	2.73	3775139	62.80	38	518358	221005	99346
**MAG.146**	88.83	2.13	3108229	61.33	111	193613	46893	28002
**MAG.147**	93.99	1.21	3839839	63.61	61	248936	103302	62948
**MAG.148**	89.86	2.83	2871840	61.59	140	127575	30924	20513
**MAG.149**	78.31	6.39	2067554	41.71	260	32699	9394	7952
**MAG.15**	89.61	5.4	7650674	69.96	1525	35885	6533	5017
**MAG.151**	97.03	0.38	2888107	43.74	24	673938	166776	120338
**MAG.17**	87.2	0	2772929	63.59	48	235042	109244	57769
**MAG.18**	91.18	4.95	3145570	43.85	376	53014	11955	8366
**MAG.2**	91.96	1.16	2212524	66.50	152	87507	21015	14556
**MAG.23**	78.74	4.69	5237741	65.04	470	59759	13157	11144
**MAG.24**	94.68	1.82	2602216	59.71	66	196546	61294	39428
**MAG.25**	82.68	1.14	3043532	63.39	422	50757	9387	7212
**MAG.27**	85.71	0	1141067	48.75	3	715151	715151	380356
**MAG.28**	91.75	0.65	4510665	60.19	105	240601	105469	42959
**MAG.29**	83.2	0.42	5032938	51.03	622	50383	10772	8092
**MAG.3**	98.39	1.52	3684554	62.34	49	388170	102647	75195
**MAG.30**	91	1.68	3201318	62.98	97	134550	55876	33003
**MAG.32**	83.19	1.87	3069251	65.54	143	80233	32839	21463

**MAG.33**	84.57	3.64	3179505	65.70	145	106484	30112	21928
**MAG.34**	80.49	0.9	3721173	59.94	17	824177	444111	218893
**MAG.38**	98.23	0.63	3315615	63.97	89	220418	59437	37254
**MAG.4**	84.32	1.71	2226814	58.61	273	31912	10895	8157
**MAG.40**	95.99	9.76	3904234	68.91	87	316549	87263	44876
**MAG.41**	85.26	2.65	3441140	45.45	47	266172	122492	73216
**MAG.42**	92.96	5.4	2986784	52.07	211	102400	21803	14155
**MAG.46**	82.64	2.66	3106609	43.04	239	91238	16781	12998
**MAG.47**	90.58	4.69	4201591	44.84	718	46473	7936	5852
**MAG.48**	89.44	1.68	1654107	44.42	98	85662	24523	16879
**MAG.49**	99.43	3.5	3529287	67.98	52	278724	102498	67871
**MAG.5**	90.87	2.72	3030024	65.15	144	80032	31692	21042
**MAG.50**	90.9	2.43	3502327	60.62	44	391221	198926	79598
**MAG.51**	93.95	0.61	2239404	40.23	12	694431	280628	186617
**MAG.53**	100	0.14	3506720	40.31	18	1498166	640255	194818
**MAG.56**	86.79	0.75	3660452	61.69	47	231609	114249	77882
**MAG.57**	97.01	3.37	3561601	66.91	221	104933	24075	16116
**MAG.59**	98.26	2.52	2739516	42.06	81	132062	49453	33821
**MAG.6**	87.43	2.64	2977572	69.95	342	69390	10874	8706
**MAG.60**	85.13	2.73	2747051	60.36	337	54557	11193	8151
**MAG.61**	91.37	3	2459140	49.64	95	144793	37335	25886
**MAG.63**	89.04	0.5	3368728	33.03	47	254293	168389	71675
**MAG.64**	93.68	1.26	2204401	43.26	58	177825	68239	38007
**MAG.67**	76.01	2.47	2536343	60.04	42	231387	147635	60389
**MAG.68**	87.92	1.08	3234704	42.29	605	39004	6856	5347
**MAG.7**	95.34	2.33	2982247	51.86	151	97004	27899	19750
**MAG.70**	93.32	1.09	3142805	62.24	133	127623	40847	23630
**MAG.71**	75.86	4.31	3218308	68.29	707	33127	5809	4552
**MAG.72**	92.61	1.85	3667257	57.93	68	371584	102460	53930

**MAG.73**	91.65	0.78	1848069	42.36	90	90841	27464	20534
**MAG.75**	93.4	5.89	2711026	31.63	66	213004	74172	41076
**MAG.78**	93.13	9.56	4570686	62.74	394	89805	17104	11601
**MAG.79**	88.58	0	3560165	69.01	62	234549	87991	57422
**MAG.8**	90.59	3.04	2393463	45.03	101	136196	35882	23698
**MAG.81**	99.32	0	2969571	38.96	53	327449	130505	56030
**MAG.82**	95.29	3.1	4388247	61.54	53	307777	149862	82797
**MAG.83**	81.6	2.47	2658818	53.06	295	54740	11488	9013
**MAG.84**	99.32	2.03	3461247	38.75	204	87587	23992	16967
**MAG.85**	91.22	3.57	3645583	41.71	61	363191	174232	59764
**MAG.87**	75.3	2.28	3074493	66.80	491	36509	7177	6262
**MAG.89**	95.35	0.39	2139168	43.34	65	159440	61853	32910
**MAG.90**	87.04	2.26	3139099	40.17	299	64523	15831	10499
**MAG.91**	82.01	2.3	2347251	50.64	199	78455	15069	11795
**MAG.93**	90.1	3.35	3008344	61.24	502	26309	7374	5993
**MAG.95**	100	2.6	3934918	58.44	41	374788	127973	95974
**MAG.96**	90.04	1.73	4096436	61.79	54	255618	127755	75860
**MAG.97**	98.96	3.28	7084418	49.82	59	419358	229546	120075
**MAG.99**	96.35	6.52	6209508	62.16	134	235958	85711	46340

Table S4 The classification of MAGs according to Genome Taxonomy Database

MAG	Domian	Phylum	Class	Order	Family	Genus	Species
**MAG.1**	Bacteria	Proteobacteria	Alphaproteobacteria	Caulobacterales	Maricaulaceae	Maricaulis	s__
**MAG.10**	Bacteria	Eremiobacterota	Xenobia	Xenobiales	JADMJV01	g__	s__
**MAG.101**	Bacteria	Proteobacteria	Gammaproteobacteria	Nitrosococcales	Methylophagaceae	Methylophaga	Methylophaga aminisulfidivorans
**MAG.102**	Bacteria	Proteobacteria	Gammaproteobacteria	Pseudomonadales	Oleiphilaceae	Marinobacter	s__
**MAG.103**	Bacteria	Proteobacteria	Gammaproteobacteria	Pseudomonadales	Nitrincolaceae	Neptuniibacter	s__
**MAG.104**	Bacteria	Actinobacteriota	Actinomycetia	Propionibacteriales	Nocardioidaceae	Nocardioides	Nocardioides sp009701885
**MAG.105**	Bacteria	Proteobacteria	Alphaproteobacteria	Sphingomonadales	Sphingomonadaceae	Sphingomonas	Sphingomonas sp002127225
**MAG.106**	Bacteria	Proteobacteria	Gammaproteobacteria	Nevskiales	Nevskiaceae	Solimonas_A	s__
**MAG.11**	Bacteria	Proteobacteria	Alphaproteobacteria	Caulobacterales	Maricaulaceae	Maricaulis	s__
**MAG.113**	Bacteria	Chlamydiota	Chlamydiia	Chlamydiales	Simkaniaceae	g__	s__
**MAG.114**	Bacteria	Deinococcota	Deinococci	Deinococcales	Trueperaceae	JAAYYF01	s__
**MAG.115**	Bacteria	Actinobacteriota	Rubrobacteria	Rubrobacterales	Rubrobacteraceae	SIRX01	s__
**MAG.116**	Bacteria	Bacteroidota	Bacteroidia	Flavobacteriales	f__	g__	s__
**MAG.117**	Bacteria	Bdellovibrionota	Bacteriovoracia	Bacteriovoracales	Bacteriovoracaceae	GCA-2712005	s__
**MAG.119**	Bacteria	Proteobacteria	Alphaproteobacteria	Caulobacterales	Maricaulaceae	Maricaulis	s__
**MAG.12**	Bacteria	Proteobacteria	Alphaproteobacteria	Sphingomonadales	Sphingomonadaceae	Sphingomonas	s__
**MAG.120**	Bacteria	Planctomycetota	Phycisphaerae	Phycisphaerales	UBA1924	UBA1924	s__
**MAG.121**	Bacteria	Proteobacteria	Gammaproteobacteria	Pseudomonadales	Spongiibacteraceae	Spongiibacter	Spongiibacter tropicus
**MAG.122**	Bacteria	Bacteroidota	Bacteroidia	Flavobacteriales	Flavobacteriaceae	Winogradskyella	s__
**MAG.123**	Archaea	Asgardarchaeota	Heimdallarchaeia	UBA460	Kariarchaeaceae	WAKA01	s__
**MAG.125**	Bacteria	Proteobacteria	Gammaproteobacteria	Burkholderiales	Burkholderiaceae	Cupriavidus	Cupriavidus campinensis
**MAG.126**	Bacteria	Proteobacteria	Gammaproteobacteria	Pseudomonadales	Pseudomonadaceae	Pseudomonas	Pseudomonas aeruginosa
**MAG.129**	Bacteria	Bacteroidota	Bacteroidia	Flavobacteriales	Flavobacteriaceae	Winogradskyella	s__
**MAG.13**	Bacteria	Actinobacteriota	Actinomycetia	Actinomycetales	Microbacteriaceae	Microbacterium	s__
**MAG.130**	Bacteria	Bacteroidota	Bacteroidia	Flavobacteriales	Vicingaceae	BRH-c54	s__

**MAG.131**	Bacteria	Actinobacteriota	Actinomycetia	Actinomycetales	f__	g__	s__
**MAG.132**	Bacteria	Bacteroidota	Bacteroidia	Flavobacteriales	Flavobacteriaceae	Arenibacter	Arenibacter algicola
**MAG.133**	Bacteria	Proteobacteria	Alphaproteobacteria	Sphingomonadales	Sphingomonadaceae	Sphingopyxis	s__
**MAG.134**	Bacteria	Proteobacteria	Gammaproteobacteria	Pseudomonadales	Pseudomonadaceae	Pseudomonas_E	s__
**MAG.135**	Bacteria	Proteobacteria	Alphaproteobacteria	Rhodobacterales	Rhodobacteraceae	Shimia	Shimia sp016019955
**MAG.136**	Bacteria	Proteobacteria	Gammaproteobacteria	Pseudomonadales	Halomonadaceae	Halomonas	Halomonas aquamarina
**MAG.138**	Bacteria	Proteobacteria	Gammaproteobacteria	DSM-19610	DSM-19610	Thiogranum	s__
**MAG.14**	Bacteria	Proteobacteria	Alphaproteobacteria	Sphingomonadales	Kordiimonadaceae	g__	s__
**MAG.140**	Bacteria	Proteobacteria	Alphaproteobacteria	Rhodobacterales	Rhodobacteraceae	Roseovarius	s__
**MAG.143**	Bacteria	Proteobacteria	Alphaproteobacteria	Caulobacterales	Caulobacteraceae	Brevundimonas	Brevundimonas aurantiaca
**MAG.144**	Bacteria	Proteobacteria	Gammaproteobacteria	SZUA-229	SZUA-229	g__	s__
**MAG.145**	Bacteria	Proteobacteria	Gammaproteobacteria	DSM-19610	DSM-19610	Thiogranum	s__
**MAG.146**	Bacteria	Proteobacteria	Gammaproteobacteria	DSM-19610	DSM-19610	Thiogranum	s__
**MAG.147**	Bacteria	Proteobacteria	Alphaproteobacteria	Rhizobiales	Hyphomicrobiaceae	Hyphomicrobium_A	s__
**MAG.148**	Bacteria	Proteobacteria	Alphaproteobacteria	Rhodobacterales	Rhodobacteraceae	WLWX01	s__
**MAG.149**	Bacteria	Proteobacteria	Alphaproteobacteria	Micavibrionales	UBA2020	g__	s__
**MAG.15**	Bacteria	Proteobacteria	Gammaproteobacteria	Burkholderiales	Burkholderiaceae	Variovorax	Variovorax sp900115375
**MAG.151**	Bacteria	Bacteroidota	Bacteroidia	Flavobacteriales	Flavobacteriaceae	Pukyongia	s__
**MAG.17**	Bacteria	Proteobacteria	Alphaproteobacteria	Rhodobacterales	Rhodobacteraceae	UBA5972	s__
**MAG.18**	Bacteria	Bacteroidota	Bacteroidia	Flavobacteriales	Flavobacteriaceae	Muricauda	Muricauda beolgyonensis
**MAG.2**	Bacteria	Actinobacteriota	Actinomycetia	Actinomycetales	Demequinaceae	Demequina	s__
**MAG.23**	Bacteria	Proteobacteria	Alphaproteobacteria	Sphingomonadales	Sphingomonadaceae	Sphingobium_A	s__
**MAG.24**	Bacteria	Proteobacteria	Alphaproteobacteria	Sphingomonadales	Sphingomonadaceae	Erythrobacter	s__
**MAG.25**	Bacteria	Proteobacteria	Alphaproteobacteria	Rhodobacterales	Rhodobacteraceae	Roseovarius	s__
**MAG.27**	Bacteria	Proteobacteria	Alphaproteobacteria	UBA1280	f__	g__	s__
**MAG.28**	Bacteria	Proteobacteria	Alphaproteobacteria	Rhodobacterales	Rhodobacteraceae	Marivita	Marivita sp017643905
**MAG.29**	Bacteria	Bacteroidota	Bacteroidia	Chitinophagales	Saprospiraceae	DT-110	s__

**MAG.3**	Bacteria	Proteobacteria	Gammaproteobacteria	Xanthomonadales	Rhodanobacteraceae	Rhodanobacter	s__
**MAG.30**	Bacteria	Proteobacteria	Gammaproteobacteria	Chromatiales	Sedimenticolaceae	g__	s__
**MAG.32**	Bacteria	Proteobacteria	Alphaproteobacteria	Sphingomonadales	Sphingomonadaceae	Rhizorhabdus	Rhizorhabdus sp004297635
**MAG.33**	Bacteria	Proteobacteria	Alphaproteobacteria	Sphingomonadales	Sphingomonadaceae	Sphingopyxis	Sphingopyxis sp001468395
**MAG.34**	Bacteria	Proteobacteria	Alphaproteobacteria	Rhizobiales	Hyphomicrobiaceae	Hyphomicrobium	s__
**MAG.38**	Bacteria	Proteobacteria	Alphaproteobacteria	Rhodobacterales	Rhodobacteraceae	Aliiroseovarius	s__
**MAG.4**	Bacteria	Proteobacteria	Gammaproteobacteria	Enterobacterales	Kangiellaceae	g__	s__
**MAG.40**	Bacteria	Proteobacteria	Alphaproteobacteria	Caulobacterales	Caulobacteraceae	Phenylobacterium	s__
**MAG.41**	Bacteria	Bacteroidota	Bacteroidia	Flavobacteriales	Flavobacteriaceae	Muricauda	s__
**MAG.42**	Bacteria	Proteobacteria	Gammaproteobacteria	UBA6429	UBA6429	JAADGZ01	s__
**MAG.46**	Bacteria	Bacteroidota	Bacteroidia	Flavobacteriales	Flavobacteriaceae	Muricauda	s__
**MAG.47**	Bacteria	Proteobacteria	Gammaproteobacteria	Enterobacterales	Alteromonadaceae	Alteromonas	Alteromonas macleodii
**MAG.48**	Bacteria	Proteobacteria	Alphaproteobacteria	UBA1280	UBA2136	UBA2136	UBA2136 sp002328405
**MAG.49**	Bacteria	Proteobacteria	Gammaproteobacteria	Xanthomonadales	Xanthomonadaceae	Pseudoxanthomonas_A	Pseudoxanthomonas_A mexicana
**MAG.5**	Bacteria	Proteobacteria	Alphaproteobacteria	Sphingomonadales	Sphingomonadaceae	Sphingosinicella	Sphingosinicella sp013911755
**MAG.50**	Bacteria	Proteobacteria	Alphaproteobacteria	Rhodobacterales	Rhodobacteraceae	HTCC2150	s__
**MAG.51**	Bacteria	Bacteroidota	Bacteroidia	Flavobacteriales	Flavobacteriaceae	MAG-120531	s__
**MAG.53**	Bacteria	Bacteroidota	Bacteroidia	Flavobacteriales	Crocinitomicaceae	SZUA-381	s__
**MAG.56**	Bacteria	Proteobacteria	Alphaproteobacteria	Rhodobacterales	Rhodobacteraceae	Pseudophaeobacter_A	Pseudophaeobacter_A gallaeciensis_A
**MAG.57**	Bacteria	Proteobacteria	Gammaproteobacteria	Burkholderiales	Burkholderiaceae	Diaphorobacter	Diaphorobacter nitroreducens
**MAG.59**	Bacteria	Proteobacteria	Gammaproteobacteria	Legionellales	Legionellaceae	g__	s__
**MAG.6**	Bacteria	Actinobacteriota	Actinomycetia	Actinomycetales	Microbacteriaceae	Microbacterium	s__
**MAG.60**	Bacteria	Proteobacteria	Gammaproteobacteria	Tenderiales	Tenderiaceae	Tenderia	s__
**MAG.61**	Bacteria	Proteobacteria	Gammaproteobacteria	Nitrosococcales	Methylophagaceae	Methylophaga	s__
**MAG.63**	Bacteria	Bacteroidota	Bacteroidia	Flavobacteriales	Flavobacteriaceae	CAU-1491	s__
**MAG.64**	Bacteria	Proteobacteria	Gammaproteobacteria	Methylococcales	Cycloclasticaceae	Cycloclasticus	Cycloclasticus sp002700385
**MAG.67**	Bacteria	Proteobacteria	Alphaproteobacteria	Rhodobacterales	Rhodobacteraceae	g__	s__

**MAG.68**	Bacteria	Bacteroidota	Bacteroidia	Flavobacteriales	Schleiferiaceae	Croceimicrobium	s__
**MAG.7**	Bacteria	Deinococcota	Deinococci	Deinococcales	Trueperaceae	g__	s__
**MAG.70**	Bacteria	Proteobacteria	Gammaproteobacteria	Nevskiales	Oceanococcaceae	Oceanococcus	s__
**MAG.71**	Bacteria	Proteobacteria	Alphaproteobacteria	Rhodobacterales	Rhodobacteraceae	Acidimangrovimonas	s__
**MAG.72**	Bacteria	Proteobacteria	Gammaproteobacteria	o__	f__	g__	s__
**MAG.73**	Bacteria	Proteobacteria	Gammaproteobacteria	Legionellales	Legionellaceae	JAALKW01	s__
**MAG.75**	Bacteria	Bacteroidota	Bacteroidia	Flavobacteriales	Flavobacteriaceae	Mesoflavibacter	Mesoflavibacter zeaxanthinifaciens
**MAG.78**	Bacteria	Actinobacteriota	Rubrobacteria	Rubrobacterales	Rubrobacteraceae	SIRX01	s__
**MAG.79**	Bacteria	Proteobacteria	Alphaproteobacteria	Sphingomonadales	Sphingomonadaceae	g__	s__
**MAG.8**	Bacteria	Proteobacteria	Alphaproteobacteria	Micavibrionales	Micavibrionaceae	UBA2705	s__
**MAG.81**	Bacteria	Chlamydiota	Chlamydiia	Chlamydiales	Parachlamydiaceae	Parachlamydia	Parachlamydia acanthamoebae
**MAG.82**	Bacteria	Proteobacteria	Gammaproteobacteria	Pseudomonadales	Alcanivoracaceae	Alcanivorax	Alcanivorax xenomutans
**MAG.83**	Bacteria	Proteobacteria	Gammaproteobacteria	Pseudomonadales	Pseudohongiellaceae	UBA9145	s__
**MAG.84**	Bacteria	Chlamydiota	Chlamydiia	Chlamydiales	Parachlamydiaceae	g__	s__
**MAG.85**	Bacteria	Bdellovibrionota	Bacteriovoracia	Bacteriovoracales	Bacteriovoracaceae	GCA-2712005	s__
**MAG.87**	Bacteria	Proteobacteria	Alphaproteobacteria	Rhizobiales	Xanthobacteraceae	Aquabacter	s__
**MAG.89**	Bacteria	Proteobacteria	Gammaproteobacteria	Legionellales	Legionellaceae	JAALKW01	s__
**MAG.90**	Bacteria	Bacteroidota	Bacteroidia	Flavobacteriales	Flavobacteriaceae	Muricauda	Muricauda sp002167435
**MAG.91**	Bacteria	Proteobacteria	Gammaproteobacteria	Nitrosococcales	Methylophagaceae	Methylophaga	Methylophaga sp014762585
**MAG.93**	Bacteria	Proteobacteria	Alphaproteobacteria	Parvibaculales	Parvibaculaceae	Tepidicaulis	Tepidicaulis marinus
**MAG.95**	Bacteria	Proteobacteria	Gammaproteobacteria	Pseudomonadales	Alcanivoracaceae	Alcanivorax	Alcanivorax sp014762765
**MAG.96**	Bacteria	Proteobacteria	Alphaproteobacteria	Rhodobacterales	Rhodobacteraceae	WKFI01	s__
**MAG.97**	Bacteria	Bacteroidota	Bacteroidia	Chitinophagales	Saprospiraceae	UBA6168	s__
**MAG.99**	Bacteria	Proteobacteria	Alphaproteobacteria	Rhizobiales	Xanthobacteraceae	Tardiphaga	s__

Table S5 The number and classification of MAGs across different sample compositions

Type	Phylum	MAG_number
**Y1&Y2&D1&D2**	Actinobacteriota	4
	Asgardarchaeota	1
	Bacteroidota	8
	Deinococcota	1
	Planctomycetota	1
	Proteobacteria	52
**Y1&Y2&D2**	Bacteroidota	1
	Bdellovibrionota	1
	Chlamydiota	1
	Proteobacteria	7
**Y1&Y2**	Bacteroidota	4
	Chlamydiota	1
	Proteobacteria	2
**D1&D2**	Actinobacteriota	2
	Chlamydiota	1
	Deinococcota	1
	Proteobacteria	2
**Y2**	Bacteroidota	1
	Eremiobacterota	1
	Proteobacteria	3
**Y2&D2**	Bacteroidota	1
	Bdellovibrionota	1
	Proteobacteria	1
**Y1&D1&D2**	Actinobacteriota	1
	Proteobacteria	1
**Y1&Y2&D1**	Bacteroidota	1
**Y1**	Bacteroidota	1
**D2**	Proteobacteria	1

Y1 and Y2 represent ballast water samples YZ202003, YZ202004 respectively. D1 and D2 represent sediment samples DN202066, DN202082 respectively.
